# Assessing primary healthcare disaster preparedness: a study in Northern Italy

**DOI:** 10.1017/S1463423624000124

**Published:** 2024-04-12

**Authors:** Alessandro Lamberti-Castronuovo, Hamdi Lamine, Martina Valente, Ives Hubloue, Francesco Barone-Adesi, Luca Ragazzoni

**Affiliations:** 1 CRIMEDIM—Center for Research and Training in Disaster Medicine, Humanitarian Aid and Global Health, Università del Piemonte Orientale, Novara, Italy; 2 Department for Sustainable Development and Ecological Transition, Università del Piemonte Orientale, Vercelli, Italy; 3 Research Group on Emergency and Disaster Medicine, Vrije Universiteit Brussel, Brussels, Belgium; 4 Department of Translational Medicine, Università Del Piemonte Orientale, Novara, Italy

**Keywords:** disaster preparedness, general practice, H-EDRM, Italy, primary healthcare

## Abstract

**Aim:**

The aim of this paper is to outline the steps taken to develop an operational checklist to assess primary healthcare (PHC) all-hazards disaster preparedness. It then describes a study testing the applicability of the checklist.

**Background:**

A PHC approach is an essential foundation for health emergency and disaster risk management (H-EDRM) because it can prevent and mitigate risks prior to disasters and support an effective response and recovery, thereby contributing to communities’ and countries’ resilience across the continuum of the disaster cycle. This approach is in line with the H-EDRM framework, published by the World Health Organization (WHO) in 2019, which emphasizes a whole-of-health system approach in disaster management and highlights the importance of integrating PHC into countries’ H-EDRM. Nevertheless, literature focusing on how to practically integrate PHC into disaster management, both at the facility and at the policy level, is in its infancy. As of yet, there is no standardized, validated way to assess the specific characteristics that render PHC prepared for disasters nor a method to evaluate its role in H-EDRM.

**Methods:**

The checklist was developed through an iterative process that leveraged academic literature and expert consultations at different stages of the elaboration process. It was then used to assess primary care facilities in a province in Italy.

**Findings:**

The checklist offers a practical instrument for assessing and enhancing PHC disaster preparedness and for improving planning, coordination, and funding allocation. The study identified three critical areas for improvement in the province’s PHC disaster preparedness. First, primary care teams should be more interdisciplinary. Second, primary care services should be more thoroughly integrated into the broader health system. Third, there is a notable lack of awareness of H-EDRM principles among PHC professionals. In the future, the checklist can be elaborated into a weighted tool to be more broadly applicable.

## Introduction

Effective disaster management draws on the resources of all actors of the healthcare system during every stage of the disaster cycle, including preparedness for unexpected events (Bayntun, 2012). The recent COVID-19 pandemic demonstrated the necessity of integrating different sectors, both within and beyond the medical field, in order to overcome challenges to public health (Parotto *et al*. 2022). Similarly, the health emergency and disaster risk management (H-EDRM) framework, published by the World Health Organization (WHO) in 2019, emphasizes the need for every level of the health system to be involved in disaster management. It particularly highlights the importance of integrating a primary healthcare (PHC) approach in countries’ H-EDRM (World Health Organization, 2019).

PHC^1^ plays an essential role in the H-EDRM framework. Its three main components (the primary care network of professionals, the people-centered approach, and the whole-of-society strategy to protect health and to reduce risks) form a fundamental backbone for effective disaster management (World Health Organization, 2018). Nevertheless, the body of literature focusing on PHC and disasters is still scant (Lamberti-Castronuovo *et al*., 2022). More research needs to be done on how to render primary care systems better prepared for disasters and on how to practically ensure the integration of PHC into national H-EDRM plans. As of yet, there is no standardized, validated way to assess the specific characteristics that render PHC prepared for disasters nor a method to evaluate its role in H-EDRM. A framework describing the key characteristics of PHC disaster preparedness has recently been proposed (Lamberti-Castronuovo *et al*., 2022).

This paper aims to describe the development of an operational checklist to assess PHC all-hazards disaster preparedness based on the H-EDRM framework and to test its use in an Italian province.

### PHC in Italy

Italy has a publicly funded national health system. Each region has exclusive responsibility for the organization, financing, and delivery of healthcare. Primary care in Italy encompasses many health services, ranging from general and rehabilitative care to family planning (Garattini and Padula, 2018). General practice is the pillar of Italian primary care and the first point of entry into the health system for the vast majority of the population’s health needs (Bonaldi *et al*., 2021). It is provided by independently contracted, self-employed physicians (general practitioners, GPs) paid on a capitation basis. Most GPs work out of small, private offices, but they can choose to go into group practice. An out-of-hours (OOH) primary care service (ie, Servizio di Continuità Assistenziale) is available throughout Italy on holidays and outside of business hours. OOH physicians are paid on an hourly basis and are under the authority of the local health department. During the COVID-19 pandemic, special additional units (ie, Unità Speciali di Continuità Assistenziale, USCA) were established as an extension of primary care services to provide ongoing, home-based health management and surveillance and timely referral to COVID-19 patients. The pandemic also prompted a reform of the Italian PHC system as a part of the National Recovery and Resilience Plan (PNRR). The reform promotes the creation of community health infrastructure which will offer basic services through an integrated and multidisciplinary organizational model of care. However, the degree of implementation of this reform is highly variable across the country (Martuscelli, 2021).

## Methodology

### Phase 1: Development of the checklist

The methodology used to develop the checklist is adapted from one study published by the WHO Emergency Medical Team Secretariat (Jensen *et al*., 2019) and from another published by the WHO Regional Office for Europe (Johansen *et al*., 2021).

The research team (ALC, HL, and MV) developed the first iteration of checklist questions using the results of an extensive literature review surveying existing characteristics of disaster preparedness at the PHC level (Lamberti-Castronuovo *et al*., 2022).

The questions were then sent to a pool of international experts with extensive experience in PHC and/or H-EDRM to solicit feedback and suggestions for making the checklist more comprehensive and effective (Annex S1). The experts’ insights were integrated into the checklist, refining it through an iterative process with the research team to produce the finalized version. This final version was then translated by the main author into Italian and adapted to reference PHC service delivery in Italy so that interlocutors would understand the items both from the language point of view and also in how different aspects of PHC (eg, workforce and service delivery) were discussed. Finally, the Italian version of the checklist was reviewed by a panel of fellow researchers and adjusted with minor changes according to their feedback, resulting in the finalized version used in the study.

### Phase 2: Testing the checklist

#### Study design and population

This is a cross-sectional study of selected primary care facilities in the Novara province of the Piedmont region in northwest Italy. The facilities were selected using purposive critical case sampling to be representative of the entire province based on the following criteria: (1) geographical (eg, facilities in city center, in hard-to-reach areas, or bordering other provinces), (2) demographic (cities versus villages), and (3) the type of primary care service offered (ie, GP versus OOH).

#### Data collection

The checklist was administered to physicians who worked in the selected facilities via interviews lasting approximately 1 h. Interviews were conducted by the main researcher between February and April 2023. Interviews occurred at the physicians’ offices so that the researcher could also conduct a visual inspection of the facilities. When an in-person meeting was not logistically feasible, interviews were conducted remotely through a videoconference system. All respondents were required to give oral informed consent prior to data collection. Interviews were conducted in Italian. When interviewees wanted to give further context for an answer, manual notes were taken by the researcher.

#### Data analysis and reporting

Data were transferred from the tool templates into an Excel spreadsheet, checked for completeness against the H-EDRM framework’s precepts, cleaned, and analyzed by the research team (ALC, HL, and MV). Given the exploratory nature of the study, a descriptive analysis was performed.

#### Ethical considerations

Sufficient details were provided at the beginning of the interview about the study’s aim and process. The data collected were anonymized, and access to data was restricted to the co-authors of this paper. Ethical approval to conduct the study was received from the Ethics Committee of the Ospedale Maggiore della Carità di Novara (Protocol 118/CE) in February 2023.

## Results

### Phase 1: Development of the checklist

A total of 10 international senior experts were recruited to provide feedback to the checklist (Annex S1). Based on the experts’ insights, the checklist’s content was optimized so that (1) all items were structured in the form of closed binary questions, (2) redundant questions were eliminated, and (3) the organization of the checklist was streamlined so that general questions prefaced more specific ones in an if/then format. For example, with regard to inquiries about multidisciplinary teams, this section was changed so that it became prefaced by an initial question: how many individuals work at the primary care facility? If the answer was only one, the rest of the section was skipped. The different sections of the checklist were then categorized according to the pillars of the H-EDRM framework, and sections related directly to policy (eg, legislation and coordination) were excluded from this publication because the study is focusing on preparedness in primary care facilities (eg, general practices). A copy of the PHC operational checklist used can be found in the supplementary material (Annex S2). Table 1 reports a breakdown of all main PHC-specific characteristics categorized according to the H-EDRM framework.

**Table 1.** PHC-specific characteristics in the PHC disaster preparedness operational checklist

	H-EDRM pillars	PHC-specific characteristics
H-EDRM functions and components at the service delivery level	1. Human resources	1.1 Multidisciplinary team 1.2 Surge capacity 1.3 Training and education 1.4 Safety and protection of workers
	2. Infrastructure and logistics	2.1 Adequate medical equipment and supplies 2.2 Appropriate infrastructure and technology 2.3 Incident response plan
	3. Health-related services	3.1 Integration of services
	4. Community engagement	4.1 Community engagement 4.2 Patient and household preparedness
	5. Information and knowledge management	5.1 Vulnerability and capacity assessment 5.2 Integration with public health functions 5.3 Research for H-EDRM 5.4 Health information system
	6. Risk communications	6.1 Coordinated communication strategies

PHC = primary healthcare; H-EDRM = health emergency and disaster risk management.

### Phase 2: Testing the checklist

A breakdown of the characteristics of the 10 primary care facilities involved in the study can be found in Table 2. All answers to the checklist questions can be seen in the supplementary material.

**Table 2.** A breakdown of the characteristics of the facilities involved in the study

Primary care facility	Type of service provided (general practice, GP; out-of-hour service OOH)	Sex	Years of activity as a PHC physician	Characteristics of the facility	Distance to the main provincial hospital
X1	GP	M	17	GP facility in a village of 1600 inhabitants (mountainous area)	20 km
X2	GP	F	8	GP facility in the main province city (city center)	2 km
X3	GP	M	11	GP facility in a village of 5K inhabitants (mountainous area)	5 km
X4	OOH	M	2	OOH in a village of 15K inhabitants (countryside)	20 km
X5	GP	F	25	GP facility in a village of 2K inhabitants (countryside)	20 km
X6	GP	F	27	GP facility in a village of 2500 (village bordering two regions)	25 km
X7	OOH	M	3	OOH in the main province city	<1 km
X8	OOH	F	10	OOH in a village of 15K (countryside)	<1 km
X9	OOH	F	10	OOH in the main province city	<1 km
X10	GP	F	11	GP facility in a village of 5K (mountainous area)	30 km

PHC = primary healthcare.

The administration of the checklist yielded information about the level of facilities’ disaster preparedness with regard to the pillars and characteristics listed in Table 1. Summaries of the insights gleaned from the checklist are found below, arranged in accordance with the H-EDRM pillars (Annex S3).

### Human resources

#### Multidisciplinary team

Among the 10 facilities in the study, 7 employed only physicians. Two facilities had physicians and an administrative assistant. One facility had a staff consisting of a physician, an administrative assistant, and a part-time nurse. The nurse managed prevention programs and undertook regular outreach initiatives, including vaccination campaigns. According to the respondent, the nurse’s presence resulted in this practice referring fewer patients to the USCA services compared with similar practices during COVID-19.

#### Surge capacity

OOH facilities had an emergency staffing plan that allowed for the transfer of physicians between their facilities. Five of the six GPs had agreements with counterparts in other facilities, which allowed them to share a health information system (HIS) and refer patients to one another in order to maintain continuity of care in case of emergencies. One GP was completely independent and, if unavailable, must refer patients to another provider on a case-by-case basis. However, due to the shortage of physicians in the area, it is still very challenging to find replacements.

#### Safety and protection of workers

All 10 respondents said that they have the necessary personal protective equipment for basic disaster response, though the volume of supplies varies between facilities. The six GPs reported experiencing significant equipment shortages during the height of the COVID-19 pandemic. Periodic health checks are required for staff of the four OOH facilities. However, as GPs are classified as self-employed workers, their personal health management is left to their own discretion.

#### Training and education

All respondents had attended courses on principles of disaster medicine during the pandemic. Prior to that, everyone recalled having attended a disaster-related course but only during their medical studies. The six GPs said they had little time for training opportunities and four still felt unprepared for disasters. OOH physicians advocated for a more robust training program on H-EDRM, specifically on triage and referral procedures.

### Health infrastructure and logistics

#### Adequate medical equipment and supplies

GP offices had neither inventoried lists of essential medications nor a surge stock of medicines. However, all GPs had verbal agreements with nearby local pharmacies in case of need. Conversely, all OOH physicians reported having both inventoried lists and a surge stock of essential medications. All respondents had professional go-bags that allowed them to write prescriptions and administer basic drugs.

#### Appropriate infrastructure

All six GP offices were located in residential units, and all four OOH facilities were found in public healthcare buildings. All facilities were accessible to people with disabilities, using ramps and/or elevators in cases where the entrance was above ground level. All facilities used a waste management system. GPs reported that no regular architectural safety assessments were performed outside of the founding of the office. OOH physicians did not know whether or not regular architectural assessments were performed. Similarly, GPs did not facilitate regular tests of their electrical, water, and air conditioning systems, while OOH physicians were unaware of any such tests at their facilities. None of the facilities had separate spaces dedicated to potentially infectious patients. Physicians in one OOH service did not have enough furniture so that the four physicians on duty had to share two computers and one examination table.

#### Incident response plan

None of the facilities had an official incident response plan with clear instructions to follow during emergency response. One respondent reported receiving official disaster preparedness instructions from governmental authorities only in preparation for big events (eg, marathons and local fairs). No formal referral pathway agreement existed between facilities and hospitals during emergencies. No memoranda of understanding existed between facilities and transportation agencies.

### Health-related services

#### Integration of services

Emergency services (eg, suturing and IV medication administration) were not performed in GP facilities. However, OOH facilities were equipped for the management of emergency procedures, including advanced life support. Initial management of acute exacerbation of chronic conditions, including mental health issues, was possible in all offices. All facilities were able to provide direct referral pathways for time-sensitive essential services (eg, contraception, abortion, and HIV testing), specialist consultations, and palliative care. However, referral pathways were not formalized, and the methods for choosing them were different at each facility.

### Community H-EDRM capacities

#### Community engagement

Only the GP facility employing a nurse had a proactive outreach initiative. The nurse scheduled regular checkups for patients and disseminated information about vaccinations. All GPs had a roster of high-risk patients who were qualified to receive primary care via house calls. However, due to physicians’ heavy workloads, intervals between visits are irregular, and house calls are done for curative rather than preventative reasons. No community engagement activities occurred in OOH facilities.

#### Patient and household preparedness

Respondents reported that discussions with patients about household preparedness strategies are rare. However, all physicians mentioned that, especially after COVID-19, encounters with patients generally include instructions on how to keep stock of essential medications. Some respondents said that before COVID-19, bulk prescriptions were uncommon, but now prescriptions are written to disburse two months’ worth of medication at a time.

### Info and knowledge

#### Vulnerability and capacity assessment

All GP facilities performed assessments based on health-related dimensions of vulnerability to identify patients that may have limited capacity to cope with the disruption of access to basic resources (eg, elderly and people with disabilities). Proactive, interdisciplinary assessments of vulnerability that include social factors and barriers to access health services were not systematically performed, and there were no disaster-specific tools used for vulnerability/capacity assessment. OOH facilities did not carry out any patient vulnerability assessments.

#### Integration with public health functions

No facilities reported the existence of clear standard procedures for integrating primary care and public health functions. During the pandemic, essential public health functions were generally performed by the USCA services, which managed contact tracing, triaging, and patient monitoring at home. The common digital platform allowed sharing of patient data among public health and USCA services. However, GPs complained that the platform was not designed to allow them to follow up on what was done by the USCA services. In the respondents’ opinions, public health services were overwhelmed and struggled to manage suspect cases, while at the same time, GPs were equally overwhelmed yet with little access to basic diagnostics and limited decision-making authority.

#### Research for H-EDRM

All 10 respondents said that they had never taken part in any research project on H-EDRM.

#### Health information system

Four out of six GPs used a cloud-based HIS. The remaining two used a local database and transferred data between computers through portable devices. All GPs had access to digital platforms for prescribing special medical equipment. Even though these platforms have streamlined processes, they are rarely integrated with each other. During the pandemic, GPs could digitally send prescriptions to pharmacies directly, and the drugs would later be delivered home to patients. This initiative was interrupted after the pandemic in almost all offices due to privacy issues. OOH facilities used digital HISs, but the information is not sharable and remains siloed in each facility’s database. There was no system for sharing electronic patient data between any of the facilities in the study and other healthcare providers.

### Risk communications

#### Coordinated communication strategies

All respondents stated they issue risk communication in accordance with directives of the public health authority. However, during the acute phase of the pandemic, there was a lack of clear communication strategies, with local health departments issuing contradictory information. GP facilities communicate with their patients through the Internet (eg, emails, messaging services, and social media). There was no specific strategy for risk communication with patients who lacked Internet access or who experienced language barriers.

## Discussion

This study describes the scientific process through which an operational checklist was developed to evaluate PHC all-hazards disaster preparedness and how this checklist was tested in a group of primary care facilities in an Italian province. The cornerstone of the methodology was based on the results of a systematic literature review on PHC disaster preparedness. These were enriched by recommendations and inputs from experts in the field of disaster management and PHC, with the ultimate goal of translating the H-EDRM framework’s precepts into a practical product.

PHC all-hazards preparedness has historically been assessed using the Hospital Safety Index (HSI) (World Health Organization and Pan American Health Organization, 2019). However, some PHC-specific characteristics are not captured by the HSI (Lapčević *et al*., 2019). Recent qualitative research calls for the development of evaluation instruments that are specific to PHC (Lamine *et al*., 2023). This study’s operational checklist focuses on characteristics peculiar to PHC during disasters, including workforce, community engagement, and service delivery, and is consistent with the body of literature on the topic (Schroeder, 2017; Burns *et al*., 2020; Frieden *et al*., 2023). Administrating the checklist in the style of an interview proved to be a useful and efficient way to obtain critical information, comments, and additional observations that enriched the assessment process. Thus, the checklist offers a practical instrument for assessing and enhancing PHC disaster preparedness and for improving coordination, planning focus, and funding allocation.

The testing of the checklist revealed three critical areas for improvement in the studied setting: (1) the composition of teams at the primary care level, (2) the lack of integration of primary care services into the broader health system, and (3) the lack of awareness on H-EDRM principles.

Although having an interdisciplinary staff in the primary care setting results in better patient outcomes (Pullon *et al*., 2016) and more effective disaster preparedness (Wyte-Lake *et al*., 2016), the primary care facilities in the study still largely operate using the traditional physician-centric care model, a finding that is consistent with previously published studies in the country (Armeni *et al*., 2014; Gualano *et al*., 2018; Garattini *et al*., 2020). The lack of multidisciplinary teams limits accessibility to primary care, incentivizing health seekers to go directly to tertiary care centers during disasters, which resulted in the overburdening of hospitals during the recent COVID-19 pandemic (Garattini et al., 2020; Johansen *et al*., 2021). The lack of multidisciplinary teams also limits the ability of primary care physicians to engage in community outreach initiatives (World Health Organization, 2021) and conduct all-hazards risk assessments to identify the most vulnerable categories of individuals. During the acute phase of the pandemic, proactive community engagement was key to granting continuity of care and improving risk communication. However, in Italy, nongovernmental organizations or community networks often stepped in to perform these functions because the primary care system was overwhelmed (Kumpunen *et al*., 2022; Parotto *et al*., 2023).

These physician-centric primary care facilities have no digital integration with one another or other parts of the health system (higher care facilities, pharmacies, public health bodies, etc.). Few facilities engaged with patients through digital means (eg, remote consultations and electronic prescriptions). Other studies corroborate this lack of integration of services (Torri *et al*., 2020; Parotto *et al*., 2022) and posit that it was a relevant factor in the spread of the pandemic in Italy (Armocida *et al*., 2020), as well as in many other countries globally (Kinder *et al.*, 2021). In order to improve the response to COVID-19, the Italian health authority took steps to make the system more integrated, building common digital platforms and instituting shared protocols and guidelines. However, these advances were seen as one-off responses to the crisis, and rather than being further developed to improve primary care preparedness strategies, they were rolled back after the crisis phase ended (Vainieri *et al.*, 2020; Kumpunen *et al.*, 2022)

A general lack of awareness of basic disaster principles emerged during the interviews. No facility performed regular hazard risk assessments in collaboration with local disaster managers, and no incident response plans were in place. No facility had ever taken part in drills with other community services. There was no awareness of H-EDRM-designated primary care tasks and competencies. These findings are consistent with previous findings in the literature that suggest that primary care professionals are seldom aware of their role in disaster management (Burns *et al*., 2019). On the same note, disaster management planners rarely include primary care professionals in H-EDRM plans (Johansen *et al.*, 2021; Kinder *et al.*, 2021). Patient outcomes would benefit from primary care cadres having a clearer definition of their roles and responsibilities in H-EDRM (Burns *et al.*, 2015; World Health Organization 2022). More training opportunities, also in the form of simulations and drills involving primary care professionals, should be organized. Moreover, there should be more scholarly research focusing on how to improve primary care and its role in H-EDRM so that effective reforms to the system can be instituted (Cegolon *et al.*, 2017; Kurotschka *et al.*, 2021).

The accuracy of the checklist’s results is confirmed by Italy’s Recovery and Resilience Plan (Governo Italiano, 2021), which also highlights the need for more interdisciplinary teams at the primary care level and further integration of the national health system. We recommend that further reform of the Italian system fully integrate PHC into the country’s H-EDRM and for more opportunities for primary care professionals, public health authorities, and disaster planners to collaborate on preparedness strategies.

## Limitations

The study presents several limitations with regard to both the methodology used for the development of the checklist and its testing. First, the checklist focuses on two out of the three main PHC components according to the WHO definition: the practical, service delivery level of PHC and the community engagement. It does not address the assessment of the policy-level aspects of the PHC integration in the H-EDRM. The policy-level checklist questions have been developed specifically for PHC and will be tested in a later study. Second, with more research, the checklist could be developed into a more robust, weighted assessment tool encompassing other aspects that may have not been captured yet (eg, ecological sustainability of health facilities) (Armocida *et al.*, 2022). Lastly, further research should focus on how other primary care professionals (eg, pharmacists) prepare for disasters and integrate their service delivery in a whole-of-health system approach with other professionals. Their role is gaining increasing relevance in H-EDRM (Watson *et al.*, 2020).

To the best of the authors’ knowledge, this is the first study targeting PHC all-hazards disaster preparedness assessment. However, this study describes the first use and testing of the checklist and focuses solely on the evaluation of the preparedness of practices in a Northern Italian province, using a limited sample size. The findings of the study cannot be generalized to all Italian primary care facilities, but the study can be replicated in other settings, including those with different healthcare models (eg, hospital-centric versus primary care-centered).

## Conclusion

This study showed the process by which an operational checklist for assessing PHC disaster preparedness was developed in order to best reflect H-EDRM precepts. It was then tested to see how well it identified areas for improvement in all-hazards disaster preparedness in a province in Northern Italy. It identified three critical areas for strengthening the role and effectiveness of PHC disaster preparedness in the province that are in line with current reforms enacted on the brink of the pandemic. Hence, this checklist may be useful to identify disaster management-specific recommendations and should be further elaborated to encompass other novel aspects (eg, environmental health). Working toward this ‘whole-of-health system’ approach in H-EDRM, deeply rooted in PHC, will make a substantial contribution to reaching health security and health for all.

## Supporting information

Lamberti-Castronuovo et al. supplementary material 1 Lamberti-Castronuovo et al. supplementary material

Lamberti-Castronuovo et al. supplementary material 2 Lamberti-Castronuovo et al. supplementary material

Lamberti-Castronuovo et al. supplementary material 3 Lamberti-Castronuovo et al. supplementary material

Lamberti-Castronuovo et al. supplementary material 4 Lamberti-Castronuovo et al. supplementary material

## Footnotes

1 In this study, PHC refers to the WHO definition of a whole-of-society approach to health, whereas primary care is defined as the first contact for health services.

## Supplementary material

To view supplementary material for this article, please visit https://doi.org/10.1017/S1463423624000124.

## References

[ref1] Armeni P , Compagni A and Longo F (2014) Multiprofessional primary care units: what affects the clinical performance of Italian general practitioners? Medical Care Research and Review 71, 315–336. 24993251 10.1177/1077558714536618

[ref2] Armocida B , Formenti B , Ussai S , Missoni E , De Marchi C , Panella M , Onder G , Mancini L , Pistis M , Martuzzi M and Barone-Adesi F (2022) Decarbonization of the Italian healthcare system and European funds. A lost opportunity? Frontiers in Public Health 10, 1037122. 36589995 10.3389/fpubh.2022.1037122 PMC9797024

[ref3] Armocida B , Formenti B , Ussai S , Palestra F and Missoni E (2020) The Italian health system and the COVID-19 challenge. The Lancet Public Health 5, e253. 32220653 10.1016/S2468-2667(20)30074-8 PMC7104094

[ref4] Bayntun C (2012) A health system approach to all-hazards disaster management: a systematic review. PLOS Currents 22, e50081cad5861d. 10.1371/50081cad5861d PMC3461969 23066519

[ref5] Bonaldi A , Celotto S , Lauriola P and Mereu A (2021) Salute per tutti: miti, speranze e certezze della Primary Health Care. Perugia: Cultura e Salute.

[ref6] Burns PL , Aitken PJ and Raphael B (2015) Where are general practitioners when disaster strikes? Medical Journal of Australia 202, 356–358. 25877108 10.5694/mja14.00477

[ref7] Burns PL , Douglas K , Hu W and Raphael B (2020) General practitioners in the field. Australian Journal of General Practice 49, 132–139. 32113212 10.31128/AJGP-08-19-5054

[ref8] Burns PL , Douglas KA and Hu W (2019) Primary care in disasters: opportunity to address a hidden burden of health care. Medical Journal of Australia 210, 297–299.e1. 30888072 10.5694/mja2.50067

[ref9] Cegolon L , Heymann WC , Lange JH and Xodo C (2017) Improving Italian general practice training: the role of academia. BJGP Open 1, bjgpopen17X100989. 10.3399/bjgpopen17X100989 PMC6169961 30564670

[ref10] Frieden TR , Lee CT , Lamorde M , Nielsen M , McClelland A and Tangcharoensathien V (2023) The road to achieving epidemic-ready primary health care. The Lancet Public Health 8, e383–e390. 37120262 10.1016/S2468-2667(23)00060-9 PMC10139016

[ref11] Garattini L and Padula A (2018) English and Italian national health services: time for more patient-centered primary care? European Journal of Internal Medicine 57, 19–21. 30279035 10.1016/j.ejim.2018.09.013

[ref12] Garattini L , Zanetti M and Freemantle N (2020) The Italian NHS: what lessons to draw from COVID-19? Applied Health Economics and Health Policy 18, 463–466. 32451979 10.1007/s40258-020-00594-5 PMC7247917

[ref13] Governo Italiano (2021) Piano Nazionale di Ripresa e Resilienza (PNRR). Roma. Retrieved 10 June 2023 from https://www.governo.it/it/approfondimento/pnrr-gli-obiettivi-e-la-struttura/16702

[ref14] Gualano MR , Bert F , Adige V , Thomas R , Scozzari G and Siliquini R (2018) Attitudes of medical doctors and nurses towards the role of the nurses in the primary care unit in Italy. Primary Health Care Research and Development 19, 407–415. 29268813 10.1017/S1463423617000846 PMC6452944

[ref15] Jensen G , Bar-On E , Wiedler JT , Hautz SC , Veen H , Kay AR , Norton I , Gosselin RA and von Schreeb J (2019) Improving management of limb injuries in disasters and conflicts. Prehospital and Disaster Medicine 34, 330–334. 31025618 10.1017/S1049023X19004242

[ref16] Johansen AS , Shriwise A , Lopez-Acuna D and Vracko P (2021) Strengthening the primary health care response to COVID-19: an operational tool for policymakers. Primary Health Care Research and Development 22, e81. 34911588 10.1017/S1463423621000360 PMC8695943

[ref17] Kinder K , Bazemore A , Taylor M , Mannie C , Strydom S , George J and Goodyear-Smith F (2021) Integrating primary care and public health to enhance response to a pandemic. Primary Health Care Research and Development 22, e27. 34109936 10.1017/S1463423621000311 PMC8220344

[ref18] Kumpunen S , Webb E , Permanand G , Zheleznyakov E , Edwards N , van Ginneken E and Jakab M (2022) Transformations in the landscape of primary health care during COVID-19: themes from the European region. Health Policy 126, 391–397. 34489126 10.1016/j.healthpol.2021.08.002 PMC8364142

[ref19] Kurotschka PK , Serafini A , Demontis M , Serafini A , Mereu A , Moro MF , Carta MG and Ghirotto L (2021) General practitioners’ experiences during the first phase of the COVID-19 pandemic in Italy: a critical incident technique study. Frontiers in Public Health 9, 623904. 33614587 10.3389/fpubh.2021.623904 PMC7888233

[ref20] Lamberti-Castronuovo A , Valente M , Barone-Adesi F , Hubloue I and Ragazzoni L (2022) Primary health care disaster preparedness: a review of the literature and the proposal of a new framework. International Journal of Disaster Risk Reduction 81, 103278.

[ref21] Lamine H , Lamberti-Castronuovo A , Singh P , Chebili N , Zedini C , Achour N , Valente M and Ragazzoni L (2023) A qualitative study on the use of the hospital safety index and the formulation of recommendations for future adaptations. International Journal of Environmental Research and Public Health 20, 4985. 36981894 10.3390/ijerph20064985 PMC10049632

[ref22] Lapčević Z , Mandić-Rajčević S , Lepić M and Jovanović M (2019) Evaluating a primary healthcare centre’s preparedness for disasters using the hospital safety index: lessons learned from the 2014 floods in Obrenovac, Serbia. International Journal of Disaster Risk Reduction 34, 436–442.

[ref23] Martuscelli C (2021) *When smaller is better: Italian health care goes local after pandemic*. Retrieved 10 June 2023 from https://www.politico.eu/article/italy-health-care-hospital-local-coronavirus-pandemic/

[ref24] Parotto E , Lamberti Castronuovo A , Della Corte F , Hubloue Y , Ragazzoni L and Valente M (2022) The COVID-19 pandemic response and its impact on post-corona health emergency and disaster risk management in Italy. Frontiers in Public Health 10, 1034196. 36388364 10.3389/fpubh.2022.1034196 PMC9659979

[ref25] Parotto E , Lamberti-Castronuovo A , Censi V , Valente M , Atzori A and Ragazzoni L (2023) Exploring Italian healthcare facilities response to COVID-19 pandemic: lessons learned from the Italian response to COVID-19 initiative. Frontiers in Public Health 10, 1016649. 36699915 10.3389/fpubh.2022.1016649 PMC9870543

[ref26] Pullon S , Morgan S , Macdonald L , McKinlay E and Gray B (2016) Observation of interprofessional collaboration in primary care practice: a multiple case study. Journal of Interprofessional Care 30, 787–794. 27797634 10.1080/13561820.2016.1220929

[ref27] Schroeder P (2017) *Canterbury Primary Response Group Emergency Plan*. Retrieved 19 June 2023 from www.primaryhealthresponse.org.nz

[ref28] Torri E , Sbrogiò LG , Di Rosa E , Cinquetti S , Francia F and Ferro A (2020) Italian public health response to the COVID-19 pandemic: case report from the field, insights and challenges for the department of prevention. International Journal of Environmental Research and Public Health 17, 3666. 32456072 10.3390/ijerph17103666 PMC7277676

[ref29] Vainieri M , Sacco Y and Ferre F (2020) A framework for assessing priority in health investments needed in Italy under the programming period 2021/2027 of the Cohesion Policy. Università Sant’Anna Pisa. Retrieved 21 June 2023 from https://www.santannapisa.it/sites/default/files/framework_thematicreports.pdf

[ref30] Watson KE , Van Haaften D , Horon K and Tsuyuki RT (2020) The evolution of pharmacists’ roles in disasters, from logistics to assessing and prescribing. Canadian Pharmacists Journal 153, 129–131. 32528591 10.1177/1715163520916921 PMC7265579

[ref31] World Health Organization (2018) Primary health care and health emergencies. Geneva: World Health Organization.

[ref32] World Health Organization (2019) Health emergency and disaster risk management framework. Geneva: World Health Organization.

[ref33] World Health Organization (2021) Multidisciplinary teams for better alignment of PHC services to meet the needs and expectations of people: Kazakhstan. Geneva: World Health Organization Regional Office for Europe.

[ref34] World Health Organization (2022) Global competency and outcomes framework for universal health coverage. Geneva: World Health Organization.

[ref35] World Health Organization and Pan American Health Organization (2019) Hospital safety index. Guide for evaluators. 2nd ed. Washington, DC: Organizacion Panamericana de la Salud.

[ref36] Wyte-Lake T , Claver M and Dobalian A (2016) Assessing patients’ disaster preparedness in home-based primary care. Gerontology 62, 263–274. 26812437 10.1159/000439168

