# Supplementary material for: Assessing primary healthcare disaster preparedness: a study in Northern Italy
Source: Prim Health Care Res Dev. 2024 Apr 12;25:e16. doi: 10.1017/S1463423624000124 (PMC11022512; doi:10.1017/S1463423624000124)
Supplement: Lamberti-Castronuovo et al. supplementary material 1 — Lamberti-Castronuovo et al. supplementary material [file S1463423624000124sup001.docx]

| **Number** | Role of the Expert | **Country** | **Field of the Expert, previous experience** | **Sex** | **Years of activity in the field of PHC or disasters** |
| --- | --- | --- | --- | --- | --- |
| **E1** | Professor in Disaster Medicine; Medical Doctor | Italy | Disaster management, academic and field experience in disaster response | M | 20 |
| **E2** | Professor of Public Health; Medical Doctor | Italy | Public Health and PHC, academic and field experience in public health integrated with PHC (e.g., vaccination campaigns, surveillance,early warning systems) | M | 40 |
| **E3** | Professor of Public Health; Epidemiologist | US | Public Health, expertise with NGOs in low income countries with Ebola, COVID-19 | M | 20 |
| **E4** | Global Health Researcher | Germany | Emergency Medicine, Assessment of Hospital Disaster Preparedness | M | 10 |
| **E5** | Global Health Researcher | Germany | Global Health, Primary Health Care, academic experience with COVID-19 and H1N1 | F | 7 |
| **E6** | Medical Doctor | Italy | Global Health, Health System Strengthening, Primary Care Physician | F | 20 |
| **E7** | Researcher | Spain | Public Health, previous work on pandemic preparedness | M | 5 |
| **E8** | Medical Doctor | Germany | Primary care physician, active academic/clinical role in care delivery to refugees | F | 15 |
| **E9** | Nurse, Global Health Researcher | Romania | Nurse, Health Delegate for NGO at the PHC level, researcher with focus on non communicable diseases in disasters | F | 15 |
| **E10** | Researcher | US | Anthropology with work experience in low  resources settings with NGOs delivering  primary care services | M | 20 |

S1: A list of all consulted experts in the iterative process. These experts were selected based on their publication records and national/international recognition as prominent professionals in the field of PHC and/or disasters.
