# Supplementary material for: Assessing primary healthcare disaster preparedness: a study in Northern Italy
Source: Prim Health Care Res Dev. 2024 Apr 12;25:e16. doi: 10.1017/S1463423624000124 (PMC11022512; doi:10.1017/S1463423624000124)
Supplement: Lamberti-Castronuovo et al. supplementary material 3 — Lamberti-Castronuovo et al. supplementary material [file S1463423624000124sup003.pdf]

ASSESSMENT OF PHC SPECIFIC COMPONENTS OF HEALTH EMERGENCY AND DISASTER RISK MANAGEMENT OPERATIONAL CHECKLIST

1. Human Resources

		Yes/No	Observations
**Multidisciplinary team**			
1	Primary care staff is composed by a **multidisciplinary team** (PhysiciansNurses Midwives Physiotherapists Social workers Psychologists Dietitians Admin staff Managers Others (if applicable) (e.g., pharmacists, dentists)		
2	Nurses take on the following roles during the disaster preparedness phase:<br>• strengthening patient education and preparedness especially for homebound individuals<br>• improving communication between staff and patients<br>• disseminating guidelines and standard procedures for disaster response<br>• answering patient questions about preparedness<br>• coordinating palliative care<br>• coordinating surge capacity during disaster response		
3	Primary care facilities have **Community Health Workers** (CHWs) who serve as intermediaries between community and PHC services. CHWs can take on: family planning services, disease surveillance, immunization, initial clinical consultations, treatment of mild infections, health education for chronic patients, education on newborn care, nutrition, proper hygiene, and safe motherhood)		
4	Primary care facilities have a **admin employees** who are in charge of office management support with health promotion, outreach, preventative measures in chronic disease management.		

		Yes/No	Observations
**Surge capacity**			
5	Primary care facilities have a clear strategy for **how to maintain staffing to continue operations** during a disaster and in its aftermath.		
6	Primary care facilities have an **emergency staffing plan** that includes workers' specific competencies, contact information, and expected availability. The plan is regularly updated.		
7	There is a list of the **competencies that non-physician roles** (nurses, community health workers) can take on during disaster response in the facility		
8	Primary care-specific emergency staffing plans are **shared among local primary care providers** and among public and private care networks to facilitate mobility of staff, thereby maximizing clinical capacity (e.g., private providers, non governmental organizations)		

		Yes/No	Observations
**Safety and protection of workers**			
9	Primary care professionals have all the **protective equipment** they need for disaster response and know how to use them, including: gloves for medical examination, gloves, surgical sterile, goggles, face shield, medical Masks, scrubs/Aprons, alcohol-based hand rub, biohazard bag, safety box for needles and sharp objects, chlorine soaps		
10	**Family support programs** are in place (e.g., child-care, flexible hours, alternative accomodation) for critical staff		
11	**Periodic health-worker wellness and exposure checks** are conducted in the preparedness phase and there is a plan to screen and test HCWs in the response phase		
12	Healthcare workers have access to **adequate services for assistance with disaster-related psychological impacts**, such as:<br>• a phone number to call for psychological assistance<br>• a designated mental healthcare professional (e.g., psychologist) to consult with		

		Yes/No	Observations
**Training and education**			
13	All primary care professionals have attended at least one course on **basic skills and concepts of DM in the last 3 years** (e.g, role of primary care professionals in emergency planning, office preparedness, human resources management during disasters, patient preparedness, mass-casualty incidents)		
14	All primary care professionals have attended at least one course on **clinical and technical skills** (i.e. triage, basic or advanced life support, bleeding control and management of disaster-specific injuries or diseases, counselling and psychological aid) **in the last 3 years**		
15	All primary care professionals have attended at least one course on **public health emergency skills** (i.e. infectious disease outbreaks, infection and prevention control standards, rational use of personal protective equipment-, surveillance/early warning, risk communication, coordination with partners, and case management for relevant public health threats) **in the last 3 years**		
16	All primary care professionals have attended at least one course on **soft skills** (e.g., teamwork, internal communication…) **in the last 5 years**		
17	All primary care professionals take part in **practical exercises** (i.e., disaster simulations), ideally in collaboration with other community institutions involved in disaster response (e.g., civil protection, hospitals, emergency medical services)		

2. Health infrastructure and logistics

		Yes/No	Observations
**Logistics, supplies**			
18	**There is an inventoried list of stocked medicines** corresponding to national list of essential drugs, ideally in digital format		
19	**There is an inventoried list of supplies and devices** and lab equipment (if applicable), ideally in digital format.		
20	Devices (electromedical equipment, life-support equipment **medical gas distribution system**) are regularly checked for functionality		
21	**A "surge stock" of medicines and supplies** (requirement for at least a month) is present and pre-positioned in areas where they are easily accessible during a disaster (e.g., in areas outside of flood risk ahead of the rainy season)		
22	**Emergency health kits or professional go-bags** (in case operations need to be conducted outside of the primary care facilities, with essential medicines and medical devices) are present in the facility		

		Yes/No	Observations
**Appropriate infrastructure**			
23	**Building integrity** (safety of foundations, structural integrity of roofs, columns, beams) is routinely checked		
24	**Physical security** of building, equipment, staff and patients is routinely assessed		
25	**Prior major structural damage/failure** of the PHC building has been assessed and the facility has been built/repaired using current safety standards		
26	**Electrical and water supply systems** are regularly tested, with a contingency plan in place for alternative sources in case of interruption		
27	**Functionality of telecommunication systems** are regularly assessed and alternative communication systems are in place.		
28	**There are areas/spaces in the primary care facilities designated for the following specific purposes:**<br>• hand-washing area for patients and for HCWs		
	• separate patient waiting area for potentially infectious patients		
	• separate consultation room for potentially infectious patients		

29	The safety of the **waste management system** (non-hazardous and hazardous solid and liquid waste system, and sharp waste if applicable) is monitored and regularly assessed		
	• Biological (if applicable)		
	• Non-biological (e.g., sharps)		
30	The condition and safety of the **heating/air-conditioning and fire protection system** (smoke detection, fire suppression) are tested every year.		

		Yes/No	Observations
**Incident response plan**			
31	Primary care facilities have an **official incident response plan** with clear instructions to follow during response and recovery actions		
32	An **incident response planning team** needs to be created with clear responsibilities within the team. The following list of roles must be included: Designated disaster coordinator, Incident first responders (health staff); First responders (non health staff); Technical (logisticians, IT)		
33	Incident response plan **contains the following information**: staff availabilities with emergency contact information; Personnel roles and lines of authority/communication; Duties assigned to personnel for emergency or disaster response and recovery; Coordination with local emergency responders; Primary care site identification strategy (sign posting of premises to help orient people toward health facility); Guidelines on donning and doffing PPE; Decontamination procedures; Procedure for recording violent episodes against health personnel.		
34	A list of all **contact information** (external stakeholder directory) needs to be created. This list needs to include: Governmental H-EDRM (State, regional, local); Health-sector institutions (e.g., public health authorities); Other non-health sector institutions (fire department, police, local NGOs and associations, local religious leaders)		
35	Facilities have an **evacuation plan** in case a disaster hits during working hours (i.e., facility access routes, emergency exits and evacuation routes, meeting points)		
36	The facility has a **business continuity plan** in case of disasters **at the facility level** (e.g., plan for modifying practice operating hours, extending consultation length)		
37	If facilities are unsafe or inoperable, there are **alternative sites** in place for continuing operations or alternative ways of delivering care (e.g., mobile clinics, telemedicine, phone consultations, remote/electronic prescriptions, e-referrals)		
38	The facility has **clear contingency plans for the provision of supplies**, equipment, and medications. These plans are drawn up in advance (e.g., a plan to ensure medication/vaccine storage if electricity fails)		
39	Capacity for **alternative sources of electricity/water and sanitation** is in place and regularly tested.		
40	Fully **functional communication system** with a backup plan for alternative means of exchanging information (e.g., radio, walkie-talkie) is in place. A single platform is identified, on which all facility and field staff are available for communication and coordination		

41	There is a designated team responsible for **rapid health-needs assessments** (in person or via telemedicine) throughout the affected community in the aftermath of a disaster		
42	There are clear guidelines on triage procedures that consider **pressing clinical needs/vulnerability factors.**		
43	Primary care facilities have cooperative arrangements with **local hospitals** regarding referral pathways to and from the facilities in case of disasters (e.g., plan for referral of suspected cases of communicable diseases)		
44	Memoranda of Understanding are in place between **facilities and ambulances/transportation** agencies to facilitate referrals to and from the primary care facilities in case of disasters		
45	**Post-mortem procedures** are known and shared among the personnel		
46	Incident response plans are regularly tested in **community-wide preparedness exercises or drills,** ideally coordinated with other agencies (e.g., hospitals, fire departments, police, schools, etc.)		

3. Health and related services

		Yes/No	Observations
**Integration of services**			
47	Minor **emergency health conditions** (e.g., minor injuries or minor infections) can be treated at the primary care level.		
	• Supplies for **management of surgical conditions** (e.g., dressing sets, bandages, gauzes, tape, gloves, scissors, general suture set, stitch removal set)		
	• Supplies for **management of medical conditions** (e.g., iv line, iv fluids, standard antibiotics)		
	• Supplies for **management of common pediatric conditions** (e.g., infant scale, tongue depressor, auriscope, ear syringe, MUAC strip)		
	• Supplies for **management of common infectious diseases** (e.g., malaria, if applicable) (e.g., ORS spoon, malaria drugs, fever-lowering drugs)		
	• Supplies for **management of maternal emergency conditions** (e.g., delivery)		
	• PHC facilities can provide first line **management of gender based sexual violence cases** (physical and mental first aid)		
	• Supplies for **management of first aid/emergency procedures** (e.g., stretcher, splints, Guedel, Ambu bag)		
	• basic **pain management**		
	• basic **mental health medications**		
48	Primary care facilities can ensure ongoing curative and preventative care for **chronic illnesses** (i.e., management of diabetes, arterial hypertension), especially **during acute exacerbations** (e.g., hypo- and hyperglycemia, hypertensive crises, asthma attacks).		
49	Primary care facilities can ensure ongoing **mental health support** (clinical encounters at the facility or remotely) with professionals (i.e., health care workers, psychotherapists, community health workers)		
50	Primary care facilities can ensure reproductive health through: the ongoing provision of **time-sensitive essential services** (e.g., contraception, abortion, MCH and pregnancy related services) **or** a direct referral pathway in place for the patient to receive the service		
51	Primary care facilities can ensure sexual health through: the provision of testing for **sexually transmitted diseases** (e.g., HIV testing) **or** a direct referral pathway in place for the patient to receive the service		
52	Primary care facilities can ensure immunization through: the ongoing provision of selected **vaccines** (e.g., COVID-19, measles, polio) **or** a direct referral pathway in place for the patient to receive the service		
53	Primary care facilities can ensure end of life care through: the ongoing provision of **palliative care for patients** (e.g., pain control) **or** a direct referral pathway in place for the patient to receive the service		

4. Community H-EDRM capacities

		Yes/No	Observations
**Community engagement**			
54	Primary care facilities run proactive **outreach initiatives** disseminating community preparedness strategies (education and promotion of awareness of different hazards using simple and understandable communication strategies)		
55	Primary care facilities run targeted **outreach initiatives** for **high-risk communities** (e.g., homebound, bedridden individuals, children with special needs, families with pets, people with hearing or visual impairment)		
56	Primary care facilities work in **collaboration with other community-based services** (e.g., non-governmental organizations, schools, places of worship, advocacy groups, pharmacies) to boost community resilience, forming **community coalitions** that undertake activities such as disaster preparedness education and participation in preparedness drills		
57	Strategies are in place to develop and implement culturally and linguistically sensitive information for people to overcome barriers to accessing information (communication strategies for people with visual/hearing impairment or with language barriers regarding for example infection prevention and control measures)		

		Yes/No	Observations
**Patient and household preparedness**			
58	Home encounters include **discussion of preparedness strategies** (e.g., smoke alarms, emergency kits, fire extinguishers, CO detectors) especially for at-risk patients (e.g., homebound)		
59	Patients are instructed on how to make an **emergency evacuation plan or an emergency go-bag** (e.g., with supplies of food and water, battery-powered radio, flashlight, emergency medications, first aid kit)		
60	Patients with chronic diseases are instructed to keep a **stock of their essential medications**		
61	Patients are taught about **key contraindications of their main medications**, on how to self-administer treatments (i.e., insulin, antibiotics), and how to use home medical devices		
62	Primary care physicians write **prescriptions in bulk** so that patients can have at least a month's worth of medication available at all times in case of an interruption in supply		
63	Patients are instructed to have **portable/digital medical history summaries** that include data about allergies, medications/doses and medical contact information		
64	Primary care professionals regularly discuss/create **preparedness plans for patients and families** (e.g., emergency contact numbers, safe meeting point in case of separation during disasters, plan to care for unattended children). Patients are instructed to share plans with friends/neighbours and other community agencies (e.g. nursing homes, schools, childcare centres).		

5. Information and knowledge management

		Yes/No	Observations
**Vulnerability and capacity analysis at the local level**			
65	Primary care professionals **undertake regular assessment** in order to identify risk factors that influence patient wellbeing (e.g., medical examination, current medications, immunisation status, physical, psychological and social functioning).		
66	Vulnerability and capacity assessment are performed through **standard tools**		
67	Vulnerability assessments are **interdisciplinary**, ideally involving medical doctors, nurses, social workers, rehabilitation therapists, pharmacists, nutrition specialists, and psychologists in order to capture all factors that contribute to individuals vulnerability		
68	Primary care facilities have a **registry of vulnerable patients by category** (homebound, bedridden, people with disabilities, etc.). This should be incorporated into primary care facilities' health information systems so that HCWs can tailor strategies for assistance during disasters		
69	Specific continuity plans need to be created for patients **dependent on medical equipment** (e.g., O2 concentrators, CPAP machines).		

		Yes/No	Observations
**Integration with public health functions**			
70	There is an agreement (e.g., memorandum of understanding) between local primary care facilities and local public health authorities to coordinate an integrated disaster response (e.g., how is the PHC system supposed to support the public health authorities during disaster response?)		
71	There is a health-surveillance strategy with an early-warning system in place (PHC providers support epidemiologic surveillance by reporting cases, by assisting with contact tracing, by providing monitoring of people in isolation)		

		Yes/No	Observations
**Research in H-EDRM at the PHC level**			
72	Have you ever performed OR participated to any research initiatives in general or specific for H-EDRM?		

		Yes/No	Observations
**Health information system**			
73	The primary care facility has a **robust cloud-based and digital health information system** (HIS) that:		
	• is centralized and remotely accessible		
	• issues a unique identification number to each patient		
	• guarantees secure, confidential, and mobile access to patient medical information		
	• allows staff to monitor patients with chronic diseases, preventing the interruption of therapies		
	• helps identify vulnerable categories of individuals		
74	HIS data is **backed up on a geographically distant server,** with records tested periodically for recoverability/completeness		
75	If the HIS is not cloud-based/digital and health records are kept on paper, files are **stored and protected from damages**		
76	Data **collection** is possible from the facilities' health information system for research purposes		
77	The primary care facility has a **fast and stable Internet connection**		
78	The primary care facility has a **robust computer network security system** (if applicable) to protect patient records		

6. Risk communications

		Yes/No	Observations
**Coordinated communication strategies**			
79	Primary care facilities ensure that their communication to the media is **in line with messaging from public health authorities**		
80	Primary care staff identifies **trusted channels of communication** and **messengers** to the community (e.g., to inform the community by sending warning messages)		
