# Supplementary material for: Assessing primary healthcare disaster preparedness: a study in Northern Italy
Source: Prim Health Care Res Dev. 2024 Apr 12;25:e16. doi: 10.1017/S1463423624000124 (PMC11022512; doi:10.1017/S1463423624000124)
Supplement: Lamberti-Castronuovo et al. supplementary material 4 — Lamberti-Castronuovo et al. supplementary material [file S1463423624000124sup004.pdf]

				GP1		GP2		GP3		GP4		GP5		GP6		GM1		GM2		GM3		GM4	
**Human resources**	**Health workforce**	1	Multidisciplinary team	☐	Group practice in a shared space but no extra staff	☑	Group practice not in a shared space with admin employee scheduling appointments remotely	☐	Digital network with other GPs. No extra staff	☐	Solo	☑	Group practice but no shared facility. No nurse. 1 Admin employee with front-office duties, prescriptions and vaccination campaigns.	☑	Group practice but no shared office. 1 Nurse. 1 Admin employee with front-office duties, prescriptions and vaccination campaigns.	☐	Only physicians. 4 during daytime and 3 during nighttime	☐	Only physicians. 2 during daytime and 2 during nighttime	☐	Only physicians. 4 during daytime and 3 during nighttime	☐	Only physicians. 2 during daytime and 2 during nighttime
		2	Nurses with specific tasks	☐	When I needed nurses, I hired them privately ad hoc	☐		☐	Concerned about nurses' lack of clear roles in PHC	☐		☐		☑	1 nurse coordinating NCD prevention programs/vaccination campaigns/ regular outreach initiatives. We had fewer contacts with USCA than my colleagues from other offices because we could do better monitoring of cases at home	☐		☐		☐		☐	
		3	Admin employees	☐		☑	scheduling appointments remotely	☐		☐		☑	Front office duties, compilation of prescriptions, vaccination campaigns	☑	Front office duties, compilation of prescriptions, medical supplies, vaccination campaigns	☐		☐		☐		☐	
		4	CHWs/Volunteers	☐		☐		☐		☐		☐		☐		☐		☐		☐		☐	
	**Surge capacity**	5	Strategy to maintain surge capacity	☑	within the group practice strategy, only patients from the list of patients registered with those GPs	☑	within the group practice strategy, only patients from the list of patients registered with those GPs	☑	within the network group strategy, generally only prescriptions	☐	working solo, recently left group practice, huge problem with replacements, shortage of HCWs	☑	within the group practice strategy	☑	within the group practice strategy, but big issue with home visits of patients residing outside of the district	☑	roster with availability, contact info	☑	roster with availability, contact info	☑	roster with availability, contact info	☑	roster with availability, contact info
		6	Staffing plan	☐		☐		☐		☐		☐		☐		☐		☐		☐		☐	
		7	Competencies for non-physicians	☐		☐		☐		☐		☐		☐		☐		☐		☐		☐	
		8	Sharing of staffing plan with other facilities (NGOs, private)	☐		☐	No, and has two different offices in two villages	☐	No, and has two different offices in two villages	☐		☐	No, and has two different offices in two villages	☐	No, and has two different offices in two villages	☐		☐		☐		☐	
	**Safety of workers**	9	Protective equipment	☑		☑		☑		☑		☑		☑		☑		☑		☑		☑	
		10	Family plans	☐		☐		☐		☐		☐		☐		☐		☐		☐		☐	
		11	OH	☐		☐		☐		☐		☐		☐		☐		☐		☐		☐	
		12	Psychology support	☐		☐		☐		☐		☐		☐		☐		☐		☐		☐	
	**Training and education**	13	Basic disaster medicine concepts	☑	during COVID-19	☑	during COVID-19	☑	during COVID-19	☑	during COVID-19	☑	during COVID-19	☑	during COVID-19	☑	during COVID-19	☑	during COVID-19	☑	during COVID-19	☑	during COVID-19
		14	Clinical competencies	☑		☑		☑		☑		☑		☑		☑		☑		☑		☑	
		15	Public health competencies	☑	during COVID-19	☑	during COVID-19	☑	during COVID-19	☑	during COVID-19	☑	during COVID-19	☑	during COVID-19	☑	during COVID-19	☑	during COVID-19	☑	during COVID-19	☑	during COVID-19
		16	Soft skills	☑		☑		☑		☑		☑		☑		☑		☑		☑		☑	
		17	Simulation and drills	☐		☐		☐		☐		☐		☐		☐		☐		☐		☐	
**Health infrastructure and logistics**	**Logistics, supplies**	18	Inventoried list of medicines	☐	no inventoried list, but only drugs as promotional drugs	☐	I try to keep as few drugs as possible	☐	no inventoried list, but only drugs as promotional tools	☐	only drugs as promotional tools	☐	only drugs as promotional tools	☐	no inventoried list	☑		☑		☑		☑	
		19	Inventoried list of supplies and devices	☐	none	☐	none	☐	none	☐	none	☐	none	☐	none	☐	none	☐	none	☐	none	☐	none
		20	Regular devices check	☐		☐		☐		☐		☐		☐		☐		☐		☐		☐	
		21	Surge stock of medicines and supplies	☐	verbal agreement with nearby pharmacy	☐	verbal agreement with nearby pharmacy	☐	verbal agreement with nearby pharmacy	☐	verbal agreement with nearby pharmacy	☐	verbal agreement with nearby pharmacy	☐	verbal agreement with nearby pharmacy	☐		☐		☐		☐	
		22	Emergency health kits	☑		☑		☑		☑		☑		☑		☑		☑		☑		☑	
	**Appropriate infrastructure**	23	Building integrity	☑	only when opening office first time	☑	only when opening office first time	☑	only when opening office first time	☑	only when opening office first time	☑	only when opening office first time	☑	only when opening office first time	☐	does not know	☐	does not know	☐	does not know	☐	does not know
		24	Physical security/Universal access	☑	elevator	☑	ground floor	☑	ground floor	☑	elevator	☑	elevator	☑	ground floor	☐	does not know	☐	does not know	☐	does not know	☐	does not know
		25	Prior damage	☑		☑		☑		☑		☑		☑		☐	does not know	☐	does not know	☐	does not know	☐	does not know
		26	Electicity and water supply system	☑	building management in charge	☑	building management in charge	☑	building management in charge	☑	building management in charge	☑	building management in charge	☑	building management in charge	☐	does not know	☐	does not know	☐	does not know	☐	does not know
		27	Functionality of telecommunication systems	☐		☐		☐		☐		☐		☐		☐	does not know	☐	does not know	☐	does not know	☐	does not know
		28	Dedicated areas	☐		☐		☐		☐		☐		☐		☐		☐		☐		☐	
		29	Waste management system	☑		☑		☑		☑		☑		☑		☑		☑		☑		☑	
		30	Air conditioning and fire protection	☑	building management in charge	☑	building management in charge	☑	building management in charge	☑	building management in charge	☑	building management in charge	☑	building management in charge	☐	does not know	☐	does not know	☐	does not know	☐	does not know
	**Incident response plan**	31	Official IRP	☐		☐		☑	official IRP only when big local fair	☐		☐		☐		☐		☐		☐		☐	
		32	IRP team	☐		☐		☐		☐		☐		☐		☐		☐		☐		☐	
		33	IRP information	☐		☐		☐		☐		☐		☐		☐		☐		☐		☐	
		34	Contact info	☐		☐		☐		☐		☐		☐		☐		☐		☐		☐	
		35	Evacuation plan	☐		☐		☑	official evacuation plan when local events happening	☐		☐		☐		☐		☐		☐		☐	
		36	Business continuity plan	☐		☐		☐		☐		☐		☐		☐		☐		☐		☐	
		37	Alternative "sites" (mobile clinics, telemedicine, e-prescriptions)	☐	e-prescription functioned only during COVID	☑		☐	e-prescription functioned only during COVID	☐		☐	e-prescription functioned only during COVID	☑		☐		☐		☐		☐	
		38	Contingency plans	☐		☐		☐		☐		☐		☐		☐		☐		☐		☐	
		39	Alternative sources	☐		☐		☐		☐		☐		☐		☐		☐		☐		☐	
		40	Communication backup	☐		☐		☐		☐		☐		☐		☐		☐		☐		☐	
		41	Rapid health needs assessment	☑		☑		☑		☑		☑		☑		☑		☑		☑		☑	
		42	Triage procedures	☑		☑		☑		☑		☑		☑		☑		☑		☑		☑	
		43	Arrangements with local hospitals	☐	very problematic	☐	usually done case by case	☐	nothing written, done case by case	☐	case by case	☐	case by case	☐	usually over the phone case by case	☐		☐		☐		☐	
		44	Arrangements with transport	☐		☐		☐		☐		☐		☐		☐		☐		☐		☐	
		45	Postmortem procedures	☑		☑		☑		☑		☑		☑		☑		☑		☑		☑	
		46	Preparedness drills	☐		☐		☐		☐		☐		☐		☐		☐		☐		☐	
**Health and related services**	**Service delivery**	47	Emergency health conditions	☑	need for extra-training,	☑	supplies are expensive and emergencies are rare	☑	need for extra-training	☑	supplies are expensive and emergencies are rare	☑	supplies are expensive and perishable, I need to pay for the stuff	☑	supplies are expensive and perishable, I need to pay for the stuff	☑	even ACLS with defibrillator	☑	even ACLS with defibrillator	☑	even ACLS with defibrillator	☑	even ACLS with defibrillator
		48	Acute exacerbations	☑		☑		☑		☑		☑		☑		☑		☑		☑		☑	
		49	Mental health support	☑	Patient's expense (private)	☑	Patient's expense (private)	☑	Patient's expense (private)	☑	Patient's expense (private)	☑	Patient's expense (private)	☑	Patient's expense (private)	☑		☑		☑		☑	
		50	Essential services	☑	Open access to family clinic, usually by phone	☑	Open access to family clinic, usually by prescription	☑	Open access to family clinic	☑	Open access to family clinic, usually by phone	☑	Open access to family clinic	☑	Open access to family clinic	☑	not authorized to write prescriptions for diagnostics	☑	not authorized to write prescriptions for diagnostics	☑	not authorized to write prescriptions for diagnostics	☑	not authorized to write prescriptions for diagnostics
		51	STDs	☑	prescription for lab exams or private lab	☑	prescription for lab exams or private lab	☑	prescription for lab exams or private lab	☑	prescription for lab exams or private lab	☑	prescription for lab exams or private lab	☑	prescription for lab exams or private lab	☑	not authorized to write prescriptions for diagnostics	☑	not authorized to write prescriptions for diagnostics	☑	not authorized to write prescriptions for diagnostics	☑	not authorized to write prescriptions for diagnostics
		52	Vaccinations	☑	Prescription	☑	Phone call to physician	☑		☑		☑		☑		☐		☐		☐		☐	
		53	Palliative care	☑	Phone call to physician in charge	☑	E-mail	☑	Web-Platform	☑	Phone call to physician in charge	☑	E-mail	☑	Phone call to physician in charge	☑	Phone call to physician in charge	☑	Phone call to physician in charge	☑	Phone call to physician in charge	☑	Phone call to physician in charge
**Community H-EDRM capacities**	**Community engagement**	54	Outreach	☐		☐		☐		☐		☐		☑		☐		☐		☐		☐	
		55	Outreach for high-risk communities	☐		☐		☐		☐		☐		☑		☐		☐		☐		☐	
		56	Collaboration with other community-based services	☐		☐		☐		☐		☑	used to organize patients groups with local NGOs but ended after COVID	☐		☐		☐		☐		☐	
		57	Communication strategies for people with barriers	☐		☐		☐		☐		☐		☐		☐		☐		☐		☐	
	**Patient preparedness**	58	Discussion of preparedness strategies	☑	happened only during COVID	☐		☑	happened only during COVID	☐		☑	happened only during COVID	☐		☐		☐		☐		☐	
		59	Emergency gobags	☐		☐		☐		☐		☐		☐		☐		☐		☐		☐	
		60	Stock of essential medications	☑		☑		☑		☑		☑		☑		☑		☑		☑		☑	
		61	Key contraindications of main medications	☑		☑		☑		☑		☑		☑		☑		☑		☑		☑	
		62	Bulk prescriptions	☑		☑		☑		☑		☑		☑		☑		☑		☑		☑	
		63	Portable medical history	☑		☑		☑		☑		☑		☑		☑		☑		☑		☑	
		64	Preparedness plans for families	☐		☐		☐		☐		☐		☐		☐		☐		☐		☐	
**Info and Knowledge management**	**Vulnerability assessment**	65	Vulnerability analysis	☑	mainly clinical risk factors	☑	mainly clinical risk factors	☑	mainly clinical risk factors	☑	mainly clinical risk factors	☑	mainly clinical risk factors	☑	mainly clinical risk factors	☐		☐		☐		☐	
		66	Assessment tools	☐		☐		☐		☐		☐		☐		☐		☐		☐		☐	
		67	Interdisciplinary assessments	☑	with social workers	☑	usually with social workers and palliative specialists	☑	with social workers	☑	with social workers	☑	with social workers	☑	with social workers	☐		☐		☐		☐	
		68	Registry of vulnerable categories	☑		☑		☑		☑		☑		☑		☐		☐		☐		☐	
		69	Continuity plans for device dependent people	☑	through device suppliers	☑	through device suppliers	☑	through device suppliers	☑	through device suppliers	☑	through device suppliers	☑	through device suppliers	☐		☐		☐		☐	
	**Integration with public health functions**	70	Written guidelines with PH for a coordinated response	☐	nothing written	☐	nothing written	☐	nothing written	☐	nothing written	☐	nothing written	☐	nothing written	☐	nothing written	☐	nothing written	☐	nothing written	☐	nothing written
		71	Effective procedures in place	☐		☐		☐		☐		☐		☐		☐		☐		☐		☐	
	**Research**	72	PHC-specific research on H-EDRM	☐	never	☐	never	☑	happened once before COVID on a project on diabetes	☐	never	☐	never	☐	never	☐	never	☐	never	☐	never	☐	never
	**Health information system**	73	Robust HIS	☐	not cloud based	☑	cloud-based, remotely accessible	☐	not cloud based	☑	cloud-based, remotely accessible	☑	cloud-based, remotely accessible	☑	cloud-based, remotely accessible	☐	no and not integrated with GPs' HIS	☐	no and not integrated with GPs' HIS	☐	no and not integrated with GPs' HIS	☐	no and not integrated with GPs' HIS
		74	Back up	☑	locally	☑		☑	locally	☑		☑		☑		☐		☐		☐		☐	
		75	Storage of records	☑		☑		☑		☑		☑		☑		☐		☐		☐		☐	
		76	Data extraction for research purposes	☑		☑		☑		☑		☑		☑		☐		☐		☐		☐	
		77	Internet connection	☑		☑		☑		☑		☑		☑		☑		☑		☑		☑	
		78	Robust network security system	☑	commercially available	☑	commercially available	☑	commercially available	☑	commercially available	☑	commercially available	☑	commercially available	☐	does not know	☐	does not know	☐	does not know	☐	does not know
**Risk communications**	**Risk communications**	79	Protocols for risk communications	☐		☐		☐		☐		☐		☐		☐		☐		☐		☐	
		80	Trusted channels of communication and messengers	☑	Emails, Whatsapp	☑	Emails, Whatsapp	☑	Emails, Whatsapp	☑	Emails	☑	Emails, Whatsapp	☐	Emails, Whatsapp	☐		☐		☐		☐	
